# Overview of Acute Ischemic Stroke Evaluation and Management

**DOI:** 10.3390/biomedicines9101486

**Published:** 2021-10-16

**Authors:** Tasneem F. Hasan, Hunaid Hasan, Roger E. Kelley

**Affiliations:** 1 Department of Neurology, Ochsner Louisiana State University Health Sciences Center, Shreveport, LA 71103, USA; roger.kelley@lsuhs.edu; 2 Hasan & Hasan Neurology Group, Lapeer, MI 48446, USA; hunaidhasan10@gmail.com

**Keywords:** thrombolytic therapy, endovascular thrombectomy, intracranial hemorrhage, antithrombotic therapy, reperfusion, functional outcome

## Abstract

Stroke is a major contributor to death and disability worldwide. Prior to modern therapy, post-stroke mortality was approximately 10% in the acute period, with nearly one-half of the patients developing moderate-to-severe disability. The most fundamental aspect of acute stroke management is “time is brain”. In acute ischemic stroke, the primary therapeutic goal of reperfusion therapy, including intravenous recombinant tissue plasminogen activator (IV TPA) and/or endovascular thrombectomy, is the rapid restoration of cerebral blood flow to the salvageable ischemic brain tissue at risk for cerebral infarction. Several landmark endovascular thrombectomy trials were found to be of benefit in select patients with acute stroke caused by occlusion of the proximal anterior circulation, which has led to a paradigm shift in the management of acute ischemic strokes. In this modern era of acute stroke care, more patients will survive with varying degrees of disability post-stroke. A comprehensive stroke rehabilitation program is critical to optimize post-stroke outcomes. Understanding the natural history of stroke recovery, and adapting a multidisciplinary approach, will lead to improved chances for successful rehabilitation. In this article, we provide an overview on the evaluation and the current advances in the management of acute ischemic stroke, starting in the prehospital setting and in the emergency department, followed by post-acute stroke hospital management and rehabilitation.

## 1. Introduction

Stroke is a major contributor to death and disability worldwide [1]. In 2019, the global prevalence of stroke was 101.5 million people, of which, 77.2 million people suffered from acute ischemic stroke (AIS) [2]. In the United States, 87% of all strokes are ischemic, while intracranial hemorrhage (ICH) and subarachnoid hemorrhage (SAH) represent 10% and 3% of all strokes, respectively [3]. The lifetime risk of overt stroke is approximately one in four by the age of 80, while the risk of silent strokes approaches 100% [4]. Before modern intervention, early mortality after stroke was almost 10% [5], while one-half of the patients developed moderate-to-severe disability and one-quarter were dependent on others [6]. Furthermore, 10% of all epilepsy, and 55% of newly diagnosed seizures in the elderly, can be attributed to stroke [7]. Despite the increasing efficacy of secondary stroke prevention, the annual stroke recurrence risk is 2.5–4.0% [8,9].

The primary therapeutic goal during acute stroke management is the timely restoration of blood flow to the salvageable ischemic brain tissue at risk for cerebral infarction [10]. Recanalization and reperfusion of the occluded vessel have been shown to reduce the infarct size and reverse neurological deficits, while a robust collateral flow helps maintain a viable ischemic penumbra [11]. The two evidence-based approaches to reperfusion are chemical thrombolysis with intravenous recombinant tissue plasminogen activator (IV TPA), and endovascular thrombectomy (EVT) with retrievable stent.

In this article, we provide an overview on the evaluation and the current advances in the management of AIS, starting in the prehospital setting and in the emergency department, with a focused discussion on reperfusion therapies, followed by post-acute stroke hospital management and rehabilitation. This review also highlights special scenarios, including strokes in people with sickle cell disease, malignant cerebral infarction, and posterior circulation large vessel occlusion. These scenarios represent important practice gaps in the field of stroke management, warranting further studies.

This review was conducted between 1 July 2020 and 10 September 2021. We searched for literature using different search terms, including: “thrombolytic therapy”; “endovascular thrombectomy”; “intracranial hemorrhage”; “antithrombotic therapy”; “stroke & functional outcome”; “stroke rehabilitation”; “stroke & sickle cell disease”; “malignant cerebral infarction”; and “posterior circulation large vessel occlusion”. We searched on PubMed (National Library of Medicine Search), MEDLINE, and Google Scholar. Peer-reviewed, evidence-based articles on the evaluation and management of AIS and related topics, published in English, were included in this review.

## 2. Stroke Classification

The Trial of Acute Stroke Treatment (TOAST) [12] classification system was developed in the early 1990s and is widely used with good interobserver agreement. It classifies ischemic strokes into five subtypes based on the underlying pathophysiological mechanism, including: (1) large artery atherosclerosis; (2) cardioembolism; (3) small vessel occlusion; (4) stroke of other determined etiology; and (5) stroke of undetermined etiology [12]. There have been further enhancements of this classification schema with the SSS-TOAST classification and the Causative Classification System [13,14].

## 3. Pathophysiological Mechanisms of Ischemic Stroke

There are several pathophysiological mechanisms underlying AIS. Thromboembolism, cardioembolism, small vessel disease, and cryptogenic are frequently observed mechanisms. Other less common mechanisms include arterial dissection, vasospasm (e.g., complication of SAH), vasculitis, vasculopathy (e.g., moyamoya disease), and hematological disorders, such as hypercoagulable states and sickle cell disease.

In AIS, arterial occlusion can arise from embolism with a cardiac source, such as atrial fibrillation, or in situ thrombus formation in the heart leading to thromboembolism. In atherosclerotic disease, AIS may result from the rupture of an extracranial plaque leading to thrombus formation, followed by the distal embolization of the thrombus to the intracranial vessels (e.g., artery-to-artery embolization). Similarly, atherosclerotic plaque rupture may also occur within the intracranial vessels, followed by in situ vessel occlusion.

Lacunar infarctions secondary to small vessel disease accounts for up to 20% of all ischemic strokes and can lead to characteristic lacunar syndromes, although the underlying mechanism for small vessel disease remains poorly understood [4]. Mechanisms, such as microatheroma with plaque rupture followed by microembolism, or the lipohyalinosis and fibrinoid necrosis of the penetrating arteries, have been suggested [15,16].

Cryptogenic strokes comprise nearly one-quarter of patients with AIS, while the term, “stroke of undetermined source (ESUS)”, is used to classify those patients having imaging findings suggestive of embolism without an identifiable etiology [17].

## 4. Diagnosis and Acute Management

The relevance of AIS detection is emphasized by the term “time is brain.” For every minute of evolution of the infarct, 1.9 million neurons, 14 billion synapses, and 7.5 miles of myelinated fibers are destroyed in patients with large vessel AIS [18]. There are various detection tools derived from the National Institutes of Health Stroke Scale (NIHSS) for use by emergency medical services in the prehospital setting for acute stroke recognition [19,20,21,22].

The NIHSS is a 42-point systematic assessment tool that should be performed within 10 min of the arrival of the patient to the emergency department. It helps the healthcare provider rapidly quantify the severity of stroke-related neurological deficit (Table 1) [23]. The NIHSS provides insight into the location (e.g., anterior versus posterior circulation, or left versus right hemisphere), and the underlying etiology (e.g., cortical versus lacunar) of the stroke. It is performed during the initial evaluation of the patient and, then, throughout the hospitalization to objectively assess the neurological status of the patient after reperfusion therapy. NIHSS scores are associated with outcome and can identify patients who are likely to benefit from reperfusion therapy or those that are at risk for developing complications from reperfusion therapy or from the stroke itself. Of note, the NIHSS tends to favor left hemispheric and anterior circulation strokes as most of the points are given to speech deficits and limb weakness, as opposed to extinction or limb ataxia, which tend to favor right hemispheric or posterior circulation strokes, respectively.

The emergency department should be notified by the emergency medical services that a patient with a suspected stroke is en route to the hospital, as this has been shown to accelerate time to thrombolysis [24]. Stratification of the level of expertise for particular hospitals, in terms of expertise with IV TPA and EVT capability, have been developed, although the value of such triage strategies has not been found to be of consistent benefit [25,26,27,28]. Mobile stroke units are also being developed in an effort to reduce the time of delivery of IV TPA [29,30,31]. In recognition of the COVID-19 pandemic and its potential effect on the delivery of care, conceptual frameworks have been proposed to address this challenge in terms of acute stroke care [32].

With the increasing use of telemedicine, patients presenting with stroke symptoms can be readily evaluated by vascular neurologists via telestroke services. Studies have shown that telestroke services are safe, improve functional outcomes, and are comparable in quality to the care that is provided in person [33,34,35]. The NIHSS assessment during a telestroke consultation is reported to be reproducible and accurate, and with favorable outcomes [36]. Of particular benefit, the practice of telestroke has increased the rate of IV TPA use from 5% to 24%, shortened the time-to-treatment (17 min versus 33 min; *p* = 0.003), and has improved acute stroke management at underserved hospitals [37,38].

Once the patient has been deemed medically stable, routine labs, including a complete blood count, comprehensive metabolic panel, and coagulation studies are drawn stat to ensure no ready contraindications to IV TPA are present, such as active bleeding, a bleeding diathesis, or a severe metabolic derangement (e.g., hypoglycemia), which could explain the presentation.

A non-contrast computed tomography (NCCT) of the head to rule out ICH or SAH, and if clinically indicated, a CT angiogram (CTA) of the head and neck, if a large vessel occlusion (LVO) is suspected, should be performed if no contraindications to iodinated contrast has been identified [39,40]. This initial neuroimaging should be performed ≤20 min upon arrival to the emergency department in at least 50% of patients who are candidates for IV TPA and/or EVT per (door-to-imaging time) recommendation [41]. A NCCT of the head can rule out ICH/SAH with >95% accuracy and may rule in the diagnosis of major stroke in two-thirds of the patients when the ischemic changes are apparent [42,43,44]. During the hyperacute stage of AIS, NCCT typically appears normal but can show subtle findings of early ischemia, including the loss of gray-white matter differentiation, cortical swelling, hypodensity from cytotoxic edema, hyperdense middle cerebral artery (MCA) sign, or sulcal effacement [45]. When performed <6-h after symptom onset, the prevalence of stroke findings on NCCT is 61%, which then progressively increases 24 h after symptom onset [45]. Of note, early evidence of infarction on NCCT may suggest a worse prognosis with poor functional outcome [45].

The Alberta Stroke Program Early Computed Tomography Score (ASPECTS) objectively quantifies, topographically, ischemic changes in the anterior circulation on NCCT of the head and identifies patients who are unlikely to recover after reperfusion therapy [46]. An ASPECTS of 10 indicates a normal NCCT, while a score of 0 indicates extensive ischemic changes. In one study, an ASPECTS ≤ 7 was associated with functional dependence and death at three months, and a predicted functional outcome with a sensitivity of 78% and specificity of 96% [46].

A CTA of the head and neck can identify extracranial and intracranial stenosis within the carotid and vertebral circulation and/or an LVO. Aortic dissection, a contraindication to IV TPA, may also be detected by a CTA [47]. In the setting of a hemorrhagic stroke, particularly SAH, a CTA can identify an intracranial aneurysm or reveal a “spot” sign as the source of bleeding in ICH [48]. At centers with 24-h magnetic resonance imaging (MRI) capabilities, after the initial NCCT of the head, an MRI and time-of-flight MR angiogram (MRA) of the brain may be obtained to avoid iodinated contrast administration in patients with contrast allergies or chronic kidney disease and to confirm the diagnosis and/or extent of the stroke. However, after ruling out ICH/SAH on the NCCT of the head, and if no other contraindications to IV TPA are recognized, administration of IV TPA should not be delayed while awaiting further imaging. 

Depending on the clinical presentation and the last known normal, advanced neuroimaging with perfusion studies, CT perfusion (CTP), diffusion weighted imaging (DWI), or MR perfusion (MRP) may be indicated [41]. Perfusion studies help define the infarcted core (irreversible ischemic tissue) and the salvageable penumbra (reversible ischemic tissue), as demonstrated by the delayed arrival of the contrast to the ischemic area. These imaging characteristics aid patient selection for EVT. One study, which correlated the infarcted areas to the salvageable areas on CTP with functional outcomes in AIS patients, found that an infarction in the following regions was associated with a worse modified Rankin Scale (mRS) score at discharge: insula ribbon (*p* = 0.023), perisylvian fissure (*p* < 0.001), motor strip (*p* = 0.007), M2 (*p* < 0.001), and M5 (*p* = 0.023) [49]. Similarly, a greater volume of an infarcted core (*p* = 0.024) was associated with a worse discharge mRS score, while a greater regional leptomeningeal collateral score (*p* = 0.004) was associated with a better discharge mRS score [49].

Stroke mimics account for 20–50% of suspected stroke presentations, depending on where the patient is being assessed and the expertise of those assessing the patient [50,51,52,53]. Stroke chameleons, in which strokes imitate other diseases, due to their atypical presentation, may also be encountered. For instance, checking the finger stick glucose is critical upon arrival as hypoglycemia (blood sugars < 50) is a common stroke mimic. If correction of the hypoglycemia does not reverse the neurological deficits, IV TPA may then be considered. It is important to recognize the potential for both stroke mimics and chameleons, particularly with atypical presentations, prior to considering reperfusion therapy (Table 2). However, when in doubt, it is probably best to proceed with IV TPA if there are not worrisome comorbidity factors that might enhance the risk of a bleeding complication [41].

## 5. Reperfusion Therapy

The goal of reperfusion therapy is to timely restore blood flow to the area of the brain that is ischemic (salvageable) but not yet infarcted. Reperfusion therapy may be comprised of IV TPA or EVT. Patients with disabling AIS are considered for reperfusion therapy (NIHSS score ≥ 5 for IV TPA; NIHSS score ≥ 6 for EVT) [54,55,56].

### 5.1. Intravenous Recombinant Tissue Plasminogen Activator

IV TPA is the mainstay of treatment for AIS within 4.5 h from a clearly defined time of symptom onset or the last known normal, when the symptom onset is unknown. IV TPA is approved by the Food Drug Administration (FDA) for the treatment of AIS within the 3-h therapeutic window. This window has been extended to 4.5 h by the American Heart Association/American Stroke Association (AHA/ASA) and the American Academy of Neurology, without FDA approval. The indications and contraindications of IV TPA are outlined in Table 3, as per the 2019 AHA/ASA guidelines, and should be discussed with the patient and/or their family prior to administration [41]. Moreover, on the basis of the ECASS-3 study, the additional exclusion criteria for the use of IV TPA previously included: age > 80-years; prior ischemic stroke plus diabetes mellitus; and anticoagulant exposure [57]. However, these criteria are no longer considered exclusions for the use of IV TPA as per the 2019 AHA/ASA guidelines [41]. 

IV TPA has shown benefit in patients with disabling strokes, regardless of the NIHSS score, but is not recommended in nondisabling strokes and/or when the NIHSS score is ≤5 [41]. The definition of “disabling”, however, differs from person to person. A patient with an NIHSS score of 2 for altered speech or vision loss has what should be considered disabling symptoms, and treatment with IV TPA would be warranted. However, an NIHSS score of 1 for sensory deficits may not be. Similarly, in our experience, a patient presenting with a transient ischemic attack, not otherwise a candidate for thrombolysis, may be considered for IV TPA in the setting of recurrent symptoms (e.g., stuttering lacunae) given its high risk of a subsequent major stroke event. Fluctuating signs and symptoms are also of concern. The patient may present with a worrisome initial NIHSS score, only to improve, but to then return to a significantly disabled condition. In such a setting, IV TPA should be considered. 

Since the benefit of IV TPA is time-dependent, it is crucial to treat patients at the earliest as this has been shown to improve outcomes [58,59]. Similarly, the decision whether to perform EVT should not delay the administration of IV TPA. A door-to-needle time <60 min in ≥50% of AIS patients should be the primary goal [41]. Nonetheless, IV TPA is reportedly underused at local hospitals where only 7.2% of AIS patients receive IV TPA within 3 h of symptom onset [60,61]. In addition, women, compared to men, are less likely to receive IV TPA, but this gap has narrowed significantly over the years [62,63]. Moreover, many AIS patients often present >4.5-h from symptom onset and are, thus, not candidates for IV TPA [64]. A common reason for delay in seeking medical care is often due to hesitation in calling 911 [65].

The administration of IV TPA within 4.5 h of symptom onset has shown to reduce functional disability, with an absolute risk reduction of 7–13% compared to placebo in various randomized controlled trials [54,57,58,66]. Beyond 4.5 h, treatment efficacy has shown to be reduced with no benefit [67]. The NINDS trial demonstrated that patients who received IV TPA were 30% more likely to have minimal or no disability at three months, with a global odds ratio (OR) for a favorable outcome of 1.7 (95% confidence interval [CI], 1.2–2.6) [54]. A follow-up pooled analysis of patients in the ATLANTIS, ECASS, and NINDS trials demonstrated that the IV TPA window could be extended to 4.5 h, with the benefits outweighing the risks of thrombolytic therapy [68]. Similarly, the ECASS-3 trial showed that those patients receiving IV TPA between 3 and 4.5 h were more likely to have favorable outcomes (OR 1.42; 95% CI, 1.02–1.98; *p* = 0.04) [57].

In the WAKE-UP trial [69], patients with AIS with an unknown time of symptom onset were considered for IV TPA when guided by a mismatch between MRI DWI and fluid attenuation inversion recovery (FLAIR) in the region of the ischemia. Specifically, the schema is an early ischemia pattern reflected as hyperintensity on the DWI but not at a state of evolution to be detected on FLAIR. In this study, significantly better functional outcome, but greater risk of ICH was noted, compared to placebo, at 90 days. The median mRS score at 90 days was 1 in the IV TPA group, and 2 in the placebo group (adjusted common OR 1.62; 95% CI, 1.17–2.23; *p* = 0.003), while the rate of symptomatic ICH was 2.0% in the IV TPA group, and 0.4% in the placebo group (OR 4.95; 95% CI, 0.57–42.87; *p* = 0.15) [69].

IV TPA should not be withheld in patients that are on antiplatelet monotherapy or dual antiplatelet therapy prior to stroke onset if they are otherwise eligible for IV TPA as symptomatic ICH, three-month mortality rates, and functional outcomes were found to be similar compared to patients that were not pretreated with antiplatelet therapy prior to stroke onset [41,70].

All patients should have two large bore IV lines placed. The dose of IV TPA is calculated as 0.9 mg/kg of the actual body weight, with a maximum dose of 90 mg. The bolus dose comprises 10% of the total dose and is administered over 1 min, while the remainder is infused over 1 h. Moreover, when there is a low suspicion for coagulopathy, IV TPA should be started prior to obtaining the results of the prothrombin time, the international normalized ratio, the activated partial thromboplastin time, and the platelet count [41]. Nonetheless, if the results do return significantly abnormal, IV TPA should be discontinued [41]. During this time, the initial stroke work-up should continue with a complete blood count, comprehensive metabolic panel, troponin, and an electrocardiogram. This work-up aids in providing vital information regarding the etiology of the stroke (e.g., new-onset atrial fibrillation, acute myocardial infarction) and may necessitate additional emergent consultations (e.g., cardiology).

Tenecteplase is another thrombolytic agent that has been modified to be more fibrin-specific and resistant to plasminogen activator inhibitor, thereby giving it a longer half-life [71]. It has been approved by the FDA for the treatment of myocardial infarction but is yet to be approved for the treatment of stroke. In a meta-analysis, tenecteplase was not superior or noninferior to standard-dose alteplase when administered as a 0.4 mg/kg single IV bolus [72]. It may be considered as an alternative means of therapy in patients with minor neurological impairment in the absence of an LVO [41]. Moreover, the TIMELESS trial is currently recruiting participants and will evaluate the 90-day efficacy and safety of tenecteplase, compared with placebo, and standard-of-care therapy in patients with AIS with symptom onset within 4.5 to 24 h [73]. Eligibility will be determined by multimodal CT or MRI and only those patients with an ICA or MCA vessel occlusion and penumbral tissue will be randomized [73].

1. Post-IV TPA Monitoring

Strict blood pressure control is vital before and after administering IV TPA to avoid the development of ICH. Prior to the administration of IV TPA, the systolic blood pressure (SBP) must be ≤185 mm Hg, and the diastolic blood pressure must be ≤110 mm Hg. If not adequately controlled, IV antihypertensives (e.g., nicardipine, labetalol, clevidipine) may be used to lower the blood pressure [41]. If the blood pressure cannot be adequately controlled within the treatment window, IV TPA should be avoided as it carries a significant risk of ICH. Moreover, after administering IV TPA, blood pressure checks should be performed every 15 min for the first 2 h, every 30 min for the next 6 h, and then hourly until 24 h after IV TPA with the goal SBP of <140 mm Hg [41]. The frequency of blood pressure checks may be further increased if the SBP is >180 mm Hg or if the diastolic blood pressure is >105 mm Hg [41].

The ENCHANTED trial evaluated 2196 IV TPA eligible patients with AIS and a SBP of ≥150 mm Hg who were randomly assigned to receive either intensive (target SBP 130–140 mm Hg within 1-h) or guideline (target SBP <180 mm Hg) blood pressure lowering treatment over 72 h [74]. While the mean SBP over 24 h showed significant difference between both the groups (intensive: 144.3 mm Hg; guideline: 149.8 mm Hg; *p* < 0.0001), the functional status at 90 days did not differ significantly between both groups (unadjusted OR 1.01; 95% CI, 0.87–1.17; *p* = 0.8702) [74]. In addition, while fewer patients in the intensive group, compared to the guideline group, had any ICH (14.8% versus 18.7%, respectively; OR 0.75; 95% CI, 0.60–0.94; *p* = 0.0137), the number of patients with any serious adverse events did not differ significantly between both groups (19.4% versus 22.0%; OR 0.86; 95% CI, 0.70–1.05; *p* = 0.1412) [74].

Blood pressure parameters in patients undergoing EVT differ from those receiving solely IV TPA, where the SBP should range between 150 and 180 mm Hg prior to reperfusion, followed by <140 mm Hg after EVT [75]. In contrast, in those patients who have not received either IV TPA or have not undergone EVT, it is important to maintain sufficient cerebral arterial perfusion during the initial 24 h to avoid recurrent strokes from hypoperfusion (e.g., permissive hypertension) [61]. The SBP is often maintained between 180–220 mm Hg, and then gradually lowered to normotensive levels over the next few days [61]. Abrupt fluctuations in blood pressure should be avoided.

After IV TPA, close monitoring in the intensive care unit or the stroke unit is necessary to assess for the development of symptomatic ICH. Vital signs and neurologic checks should be performed frequently to monitor for any neurological decline. Moreover, antithrombotic agents should be held for the first 24 h after administering IV TPA and should be (re-)initiated after obtaining a follow-up NCCT of the head if no hemorrhagic conversion of the infarct is noted [41]. In addition, intraarterial pressure catheters, indwelling bladder catheters, and nasogastric tubes should be avoided during the first 24 h, if possible, to prevent the risk of any inadvertent bleeding [41].

2. Potential Complications of IV TPA

IV TPA is not without potential complications. The most feared complication is symptomatic ICH, reported in 6.4% of patients in the NINDS trial, while asymptomatic ICH, major systemic hemorrhage, and angioedema may also occur [54]. A sudden neurological decline, including altered levels of consciousness, severe headache, nausea, and/or vomiting may indicate ICH. If the infusion is still ongoing, it must be discontinued immediately, and a stat NCCT of the head must be obtained to assess for ICH [41]. Laboratory tests including a complete blood count with platelet count, blood type and crossmatch, a prothrombin time, an activated partial thromboplastin time, and a fibrinogen level should be obtained. Potential agents for IV TPA reversal may include cryoprecipitate, aminocaproic acid, or tranexamic acid [41,76,77,78,79]. IV cryoprecipitate 10-units may be infused over 10 to 30 min, and repeated, to maintain a serum fibrinogen level of 150–200 mg/dL. In addition, supportive therapy is warranted and includes the appropriate management of the blood pressure, intracranial pressure, cerebral perfusion pressure, and blood glucose. Neurosurgery and hematology services should be consulted urgently [41]. Furthermore, systemic bleeding may occur from catheter sites, or may manifest as gum bleeding or ecchymoses, but this does not warrant discontinuation of IV TPA.

Angioedema occurs in nearly 8% of the patients receiving IV TPA [80,81,82]. The risk is increased in patients on an angiotensin converting enzyme inhibitor. While mild orolingual angioedema is most common, severe cases can occur and may cause airway obstruction warranting endotracheal intubation [41,81,83,84]. With angioedema, intubation may be difficult as it often distorts the normal orolingual anatomy. Nasotracheal intubation or cricothyrotomy may be considered but are reserved for severe cases as they carry a higher risk for bleeding after IV TPA [41]. While evaluating for patent airway, IV TPA should be discontinued and IV methylprednisolone 125-mg, IV diphenhydramine 50-mg, and IV famotidine 20-mg should be administered [41]. Additionally, if the patient is on an angiotensin converting enzyme inhibitor, it should be discontinued. In patients with refractory angioedema, icatibant may be of benefit [41].

3. Bridging Therapy

There has been some conflicting data in reference to combining IV TPA with EVT, in a bridging format, as there have been questions raised about the benefit compared to the potential lower efficacy of EVT because of the delay in initiation associated with the preliminary IV TPA infusion. Prior studies have shown that IV TPA may be ineffective in dissolving large clots and the re-occlusion of blood vessels after initial recanalization with IV TPA is common [85,86]. Thus, EVT has emerged as a more effective approach to LVOs in accessible vascular regions, including both the anterior and posterior circulations. Two recent studies have supported bridging therapy. Ahmed et al. [87] reported improved outcomes, both in terms of functional independence as well as in mortality, at three months, with the combination. This was supported as well by a meta-analysis study [88], in which there were similar outcomes at three months with or without bridging therapy, but the nonbridging patients had lower rates of successful recanalization.

### 5.2. Endovascular Thrombectomy

The use of EVT has revolutionized the field of AIS. The field of endovascular stroke management started to emerge after the approval of the first generation MERCI device by the FDA. This device was utilized in the MERCI and MULTI-MERCI trials, forming the groundwork for further research within the field of EVT [89,90]. Of interest, in 2013, there were three landmark trials, SYNTHESIS, MR RESCUE, and IMS III, which failed to demonstrate any benefit of EVT compared to standard medical therapy [91,92,93]. Lessons learned from these trials led to the design of more stringent patient selection criteria. In 2015, EVT became the standard of care after five prospective trials demonstrated the benefit of EVT compared to medical management alone in select patients with AIS [94,95,96,97,98]. The HERMES meta-analysis included the trials, MR CLEAN, ESCAPE, REVASCAT, SWIFT PRIME, and EXTEND IA, and also found that EVT was beneficial in patients with AIS from a proximal anterior circulation occlusion [99].

On the basis of these trials, the decision for EVT was strictly based on time criteria, where patients presenting within 12 h from symptom onset were eligible for EVT, and thereby excluding patients with wake-up, unwitnessed, or late presenting strokes. The successful results of the DAWN [100] and the DEFUSE-3 [101] trials represent a paradigm shift in the field of EVT for the management of AIS from LVO. These trials have extended the therapeutic window for EVT from 6 to 8 h of symptom onset to 24 h after the last known normal. Of pertinence, patients presenting with wake-up strokes may now be eligible for EVT based on the DAWN and the DEFUSE-3 trials [100,101]. These landmark EVT trials have been summarized in Table 4.

The DAWN trial [100] selected patients for EVT based on the presence of salvageable brain tissue, as determined by MRI DWI or CTP, and evidence of clinical mismatch, defined as the severity of neurological deficits compared to the volume of infarcted brain. The treatment group was comprised of EVT plus standard care, while the control group was comprised of standard medical therapy alone. At 90 days, the mean utility-weighted mRS score was 5.5 in the treatment group, compared to 3.4 in the control group, while the rate of functional independence was 49% in the treatment group, and 13% in the control group. No significant difference was noted between the treatment and control groups for symptomatic ICH (6% versus 3%; *p* = 0.50) or mortality (19% versus 18%; *p* = 1.00). The rate of recanalization at 24 h was 77% in the treatment group, compared to 39% in the control group. The number-to-treat to achieve lower disability and functional independence at 90 days was 2 and 2.8, respectively [100].

The DEFUSE-3 trial [101] selected patients for EVT between 6 and 16 h from their last known normal, and who had salvageable brain tissue. Patients with a proximal MCA or ICA occlusion, initial infarct size < 70 mL, and a ratio of the volume of ischemic tissue on perfusion imaging to infarct volume of ≥1.8, were randomized to either the treatment group (EVT plus standard medical therapy) or the control group (standard medical therapy alone) [101]. The treatment group was found to have a favorable shift in the distribution of functional outcomes on the mRS at 90 days (OR 2.77; *p* < 0.001), with a higher percentage of patients with functional independence (mRS score 0–2) when compared to the control group (45% versus 17%; *p* < 0.001). The 90-day mortality rate was 14% in the treatment group, and 26% in the control group (*p* = 0.05), while no significant difference was noted for symptomatic ICH (7% versus 4%; *p* = 0.75) or serious adverse events (43% versus 53%; *p* = 0.18) between both groups, respectively [101].

The SELECT-2 trial [102] is currently recruiting and will evaluate the efficacy and safety of EVT with stent retrievers (Trevo^®^, Solitaire^®^, and EmboTrap^®^), compared to standard medical therapy alone, in patients with AIS due to a distal ICA and MCA M1 LVO who present with a large core (≥50 cc) on the NCCT of the head (ASPECTS 3–5) and/or advanced perfusion imaging (regional cerebral blood flow < 30% on CTP or apparent diffusion coefficient < 620 on MRI), and are treated within 24 h from the last known normal.

In a recent study, the prevalence of salvageable tissue ≥16-h from the last known normal after an AIS from an LVO was estimated and the effectiveness of EVT was evaluated. Of the 150 patients, 16% who underwent EVT had better odds of a 90-day mRS score of 0–2 (adjusted OR 11.08; 95% CI, 1.88–108.60) and a 90-day mRS score shift (common adjusted OR 5.17; 95% CI, 1.80–15.62) [103]. Similarly, in a subgroup of 109 patients who were 24 h from their last known normal, EVT was associated with a favorable mRS score shift (common adjusted OR 10.54; 95% CI, 2.18–59.34) [103]. Thus, carefully selected patients presenting ≥16-h to 10 days from their last known normal with an anterior circulation LVO may benefit from EVT [103].

In another study, clinical outcomes in the elderly (≥80 years) with AIS treated with EVT were explored [104]. Elderly patients were found to have worse functional outcomes (adjusted common OR for mRS score shift toward better outcome 0.31; 95% CI, 0.24–0.39) and increased mortality (51% versus 22%; adjusted OR 3.12; 95% CI, 2.33–4.19) as compared to younger patients. However, successful reperfusion was more strongly associated with a shift toward good functional outcomes in this subgroup of elderly patients [104].

Appropriate patient selection is essential for improving the outcomes of EVT. An NIHSS score > 6, or a lower score with significant disability, such as aphasia, should be considered for EVT [19,105]. The patient’s premorbid condition is also crucial when considering EVT. Many landmark trials on EVT were comprised of patients with a premorbid condition with an mRS score of 0–1 or 0–2 [94,95,96,100,101]. For example, a patient who is wheelchair-bound and/or unable to the perform activities of daily living at baseline may not be a candidate for EVT as the risk of the procedure would outweigh its benefit. Moreover, the imaging parameters considered favorable include: ASPECTS of 6–10; a significant area of mismatch volume (≥15 mL) or mismatch ratio (≥1.8) on CTP or MRP; a core infarct of ≤70 mL; and evidence of an LVO in the proximal anterior circulation (M1 and cervical and intracranial ICA), with good collaterals on CTA/MRA [100,101,106]. If the patient presents within 24 h from their last known normal and have met the above criteria, they should be considered for EVT without delay. Nonetheless, pursing EVT outside the established parameters is clinician-dependent with other factors, among centers, that may influence the decision for EVT [105].

While EVT represents a milestone in the management of AIS from an LVO, there still exists gray areas in the field of EVT. The majority of the EVT trials have enrolled patients with an LVO in the anterior circulation, including ICA and proximal MCA occlusions. However, the role of EVT within the distal or posterior circulation remains unclear and warrants further study.

## 6. Post-Stroke Antithrombotic Therapy

### 6.1. Antiplatelet Therapy

Antiplatelet therapy is held in AIS patients who receive IV TPA until the 24-h NCCT of the head shows no hemorrhagic conversion of the infarct. Thereafter, aspirin is routinely started, if there are no contraindications, to all ischemic stroke patients, with a typical dosing of 160 to 325 mg a day based upon the CAST [107] and IST [108] studies. For those patients who have failed aspirin therapy since having a recurrent stroke while compliant on aspirin, monotherapy with clopidogrel may then be considered. Based upon information derived from the CHANCE [109] and the POINT [110] studies, patients with transient ischemic attack and minor stroke are often treated with a 21-day course of dual antiplatelet therapy (aspirin and clopidogrel). The initial aspirin dose may be 325 mg followed by 81 mg a day, and the initial clopidogrel dose may be 300 mg followed by 75 mg a day. Beyond three weeks of dual antiplatelet therapy, the potential risks start to outweigh the potential benefits, based upon the CHARISMA [111] and the MATCH [112] studies, as well as the CHANCE [109] and the POINT [110] studies. Thus, monotherapy with either aspirin or clopidogrel is continued thereafter.

### 6.2. Anticoagulant Therapy

Atrial fibrillation is an important risk factor for cardioembolic ischemic stroke, silent infarctions, and transient ischemic attacks [113,114,115]. Strokes resulting from atrial fibrillation tend to be severe, resulting in greater disability and mortality than those patients with strokes from a different etiology [116,117,118]. Despite anticoagulant therapy, such patients may still have recurrent strokes, presumably from other mechanisms [119]. In a patient with atrial fibrillation who is not anticoagulated, the risk for recurrent stroke is 3–5% within the first few weeks after the initial event [108,120], followed by 12% annually in the first 2–3 years [121,122].

The CHA_2_DS_2_VASc score [123] calculates the risk of a thromboembolic event at one year in a nonanticoagulated patient with atrial fibrillation. A score of 0 is considered low risk and may not require treatment, while a score of 1 is considered low-moderate risk and may warrant treatment with antiplatelet or anticoagulant therapy. A score of ≥2 is considered moderate-high risk, and the patient should be anticoagulated if no contraindications exist. The HAS-BLED score [124] is utilized in a patient that may benefit from anticoagulant therapy but may also harbor a high risk for bleeding complications, such as frequent falls. This score estimates the risk of major bleeding and assesses the risk-benefit of anticoagulation for atrial fibrillation.

The time of (re-)initiation of anticoagulation is predicated on the extent of the infarct and whether there is associated secondary hemorrhagic transformation of the infarct [125,126]. Current recommendations are based on consensus opinion [41,127], observational studies [128,129,130], and cohort studies [131]. The 2020 European Society of Cardiology guidelines on the management of atrial fibrillation recommends (re-)initiation of anticoagulation at the earliest, often within two weeks [132], while the 2019 AHA/ASA guidelines recommend (re-)initiation of anticoagulation anywhere between 4 and 14 days from the acute stroke [41]. However, a European Stroke Organization expert consensus reports that recommendations for the optimal timing for anticoagulation initiation in patients with AIS could not be made [133]. Moreover, the 2021 European Heart Rhythm Association practical guide provides timeframes of when to (re-)initiate anticoagulation based on expert opinions, such that after a transient ischemic attack, anticoagulation may be continued or started the next day after excluding ICH on imaging [126,127,134]. In patients with mild stroke, anticoagulation may be (re-)initiated ≥ 3 days after AIS, while in moderate and severe strokes, anticoagulation may be (re-)initiated ≥ 6–8 days and ≥12–14 days after AIS, respectively, once excluding secondary hemorrhagic transformation on repeat imaging [127,134]. In the interim, antiplatelet therapy should be continued until the (re-)initiation of anticoagulation, and anticoagulation should only be (re-)initiated if the stroke size is not expected to substantially increase the risk of secondary hemorrhagic transformation [127,134]. When secondary hemorrhagic transformation is present, anticoagulation may be (re-)initiated anywhere between 3 and 28 days [127,134]. Nonetheless, several randomized trials (e.g., ELAN [NCT03148457], OPTIMAS [NCT03759938], TIMING [NCT02961348], START [NCT03021928], AREST [NCT02283294]) investigating early versus late (re-)initiation of anticoagulation after AIS are currently ongoing [129].

## 7. Post-Acute Stroke Hospital Management

All new and recurrent stroke patients, including high-risk transient ischemic attacks, irrespective of reperfusion therapy, are admitted to a dedicated stroke unit for close neurologic and cardiac monitoring and to complete the stroke work-up. Patients treated in a dedicated stroke unit have shown to do better than those treated in the general inpatient ward, and are more likely to be independent and live at home at one-year post-stroke [135,136,137,138,139,140]. In one study, treatment provided in a stroke unit significantly reduced hospital mortality (OR 0.50; 95% CI, 0.34–0.74), case-fatality (OR 0.45; 95% CI, 0.28–0.71), six-month mortality (OR 0.57; 95% CI, 0.39–0.82), one-year mortality (OR 0.59; 95% CI, 0.42–0.84), and the rate of discharge to nursing homes (OR 0.61; 95% CI, 0.38–0.98) [139].

After the acute phase, completing the stroke work-up is crucial in identifying the underlying stroke etiology in order to effectively implement secondary stroke prevention strategies. A cardiovascular examination and an electrocardiogram should be routinely performed in all stroke patients. It allows for the recognition of acute myocardial ischemia and/or cardiac arrhythmia, such as atrial fibrillation, which would warrant an urgent cardiology consultation [141]. It is also important to recognize that the acute cerebral infarction can stress the heart and promote ischemia as well as arrhythmia. Right hemispheric infarctions have a high risk of arrhythmias caused by disturbances in the sympathetic and parasympathetic nervous system function [142,143,144,145].

Transthoracic echocardiogram is vital in the evaluation of stroke etiology. It can determine the percentage of ejection fraction, the degree of anterior wall motion abnormalities, and the presence of valvular disease or a thrombus [146]. While it should be routinely performed in stroke patients < 50 years of age, its use in older patients (>70 years) can result in nonspecific findings [146]. Nonetheless, the value of a transthoracic echocardiogram has been much debated. While it may lead to changes in management, the changes are often not supported by data that outcome is improved. Moreover, a transesophageal echocardiogram is superior to a transthoracic echocardiogram in identifying high-risk sources, such as thrombus formation or a patent foramen ovale, which serves as a potential conduit for cerebral embolism [147,148,149]. The 10-point risk of paradoxical embolism (RoPE) score has been used to select patients for patent foramen ovale closure in cryptogenic strokes; however, its usefulness is still being debated [150]. Furthermore, a transesophageal echocardiogram may influence the use of oral anticoagulation in patients with stroke of undetermined etiology [148,151].

Carotid ultrasound is a noninvasive imaging technique for the evaluation of extracranial carotid artery lesions which are implicated in patients with AIS [152]. On ultrasound, plaques are characterized as echogenic, calcified, or hypoechoic. They are further characterized by intraplaque hemorrhage, surface ulcerations, and the percentage of stenosis [153]. Moreover, a transcranial Doppler can be used to detect cerebral emboli from an in situ thromboembolic source [154]. The detection of asymptomatic cerebral embolic signals may be a potential marker of stroke risk. Transcranial Doppler may help identify patients at high risk for recurrent stroke, which could impact the choice of antithrombotic medication [155].

Electroencephalogram detects summated responses from the pyramidal neurons residing predominantly in cortical layers 3, 5, and 6, generating graded postsynaptic excitatory and inhibitory potentials [156]. Electroencephalogram changes have shown to occur within 5 min of AIS [157,158]. Quantitative electroencephalogram monitoring parameters are also used to detect various patterns of ischemia, such that early and subtle ischemia may result in a regional alpha-beta/theta-delta power ratio, while moderate to severe acute hemispheric ischemia may result in widespread polymorphic delta activity in the involved hemisphere, maximally seen in the temporal and frontotemporal regions [159,160,161]. Moreover, large subcortical ischemic strokes may result in both focal or generalized intermittent rhythmic theta and delta activity, while lacunar infarcts typically show normal or subtle focal theta activity [162,163].

Hemoglobin A1c, low-density lipoprotein, and thyroid-stimulating hormone help identify the modifiable risk factors of stroke. Toxicology or drug screening, pregnancy tests, and blood alcohol levels are additional studies that should be obtained if patient history is uncertain, or examination findings are concerning. Arterial blood gas levels should be drawn if there is concern for hypoxia.

## 8. Stroke Rehabilitation

In the modern era of acute stroke care, more patients will survive with varying degrees of disability post-stroke [164]. A comprehensive stroke rehabilitation program is critical to optimize post-stroke outcomes. Understanding the natural history of stroke recovery and adapting a multidisciplinary approach will lead to successful rehabilitation [164].

During stroke rehabilitation, motor impairment is commonly encountered [165]. Other deficits include language and articulation dysfunction, difficulty swallowing, vision changes, sensory deficits, and cognitive impairment [165]. While there is scarce evidence for impairment-focused therapies, there is strong evidence for task and context-oriented rehabilitation, facilitating the natural course of functional recovery [166,167,168]. In addition, adaptive strategies, which compensate for impaired function, highly correlate with improved functional recovery [169,170,171].

Stroke rehabilitation is a cyclical process involving: (1) assessment—detecting and quantifying the patient’s deficits; (2) goal setting—defining clinically appropriate goals for improvement; (3) intervention—intervening in the achievement of goals; and (4) reassessment—reevaluating progress for set goals [172]. Rehabilitation should be initiated within the first 24–48 h post-stroke and should involve short frequent training sessions to improve outcome [173,174]. The VECTORS trial, which studied the amount of therapy and motor improvement after stroke, suggested that more therapy did not translate into better outcomes [175]. Similarly, the AVERT trial found that very early mobilization (<24-h after stroke), with frequent and higher doses of therapy compared to standard therapy, was associated with reduced odds of a favorable outcome at three months, but at twelve months, health-related quality of life was similar between both groups [176]. A multidisciplinary team care approach, with patient and/or family engagement and motivation, is strongly supported and has been linked to better rehabilitation outcomes [177,178].

Pharmacological agents may also promote post-stroke recovery. While selective serotonin reuptake inhibitors have shown benefit in patients with post-stroke depression, the FLAME trial, comparing fluoxetine 20-mg daily to placebo, 5–10 days post-stroke, also reported improved motor recovery as a result of neuroplasticity [164,179]. However, two subsequent trials failed to demonstrate any benefit post-stroke. In the EMOTION trial, there was no significant reduction in moderate to severe depression in patients with AIS when treated with escitalopram [180], while in the FOCUS trial, there was no meaningful improvement in functional outcomes when treated with fluoxetine 20-mg daily for six months after stroke despite improvements in depression. The results of the FOCUS trial do not support the routine use of fluoxetine for either prevention of post-stroke depression, or to promote functional recovery [181].

## 9. Special Scenarios

### 9.1. Sickle Cell Disease

Strokes due to sickle cell disease are unique because of the heterogeneous etiology affecting the management approach. Strokes in children tend to present with infarction, while strokes in adults often present with hemorrhage. The most common subtype of cerebral infarction is the border-zone stroke type, which occurs between the anterior cerebral artery and MCA territories. Systemic stressors cause a lack of cerebrovascular reserve with perfusion failure, which occurs distal to a fixed stenosis. Moreover, other mechanisms of stroke in sickle cell disease are the occlusion of large- and medium-sized vessels, artery-to-artery embolism and fat embolism, and thrombosis and/or the clumping of sickled erythrocytes. Conversely, in adults with hemorrhagic strokes, aneurysmal disease and fragile moyamoya-type collaterals have been demonstrated. Moreover, the identification of sickle cell disease patients most at risk for AIS has been significantly enhanced by the use of serial transcranial Doppler studies.

Blood transfusion therapy is vital for the prevention of strokes in sickle cell disease patients [182,183,184]. Blood transfusions lead to improvement in the oxygen saturation through increasing the arterial oxygen pressure and hemoglobin–oxygen affinity and, thereby, reducing the sickling of cells [185]. The use of blood transfusion for primary stroke prevention has shown a >80% reduction in the stroke rate, such that the baseline risk for strokes in sickle cell disease patients is nearly 70%, but with periodic transfusions, the risk may be reduced to as low as 13% [186,187].

A potential curative treatment for sickle cell disease is bone marrow transplantation. In patients with a history of symptomatic cerebrovascular event status post bone-marrow transplantation, there have been no reported stroke recurrences with the notable regression of cerebral vasculopathy [188,189,190]. Moreover, hydroxyurea is an alternative to transfusion therapy for secondary stroke prevention. Hydroxyurea is a nitric-oxide donor, contributing to its clinical effect [191]. The mechanism of action is through the stimulation of the production of fetal hemoglobin which, thus, inhibits the polymerization of deoxygenated sickle hemoglobin [192].

The SWiTCH trial demonstrated that transfusion and chelation therapies were better management approaches for children with sickle cell disease, stroke, and iron overload [193]. The TWiTCH trial, however, reported that in high-risk children with abnormal transcranial Doppler velocities, who were four years post-transfusion, with no severe MRA vasculopathy, hydroxyurea therapy could be effectively substituted for chronic transfusions [194].

### 9.2. Malignant Cerebral Infarction

Malignant cerebral infarction is a large MCA infarction with or without the involvement of the ipsilateral anterior and/or posterior cerebral artery territories. It presents with acute brain swelling, 48 to 72 h after the stroke, with significantly elevated intracranial pressure and with the potential risk for cerebral herniation. The development of malignant cerebral infarction can be predicted by ischemia affecting more than two-thirds of the MCA territory, with a sensitivity of 91% and a specificity of 94% [195].

Management of malignant cerebral infarction involves: (1) medical therapy with osmotherapy; (2) intracranial pressure monitoring; and (3) surgical decompressive craniectomy, as clinically indicated. The first-line osmotherapy for cerebral edema are mannitol and hypertonic saline. Mannitol administration should be weight-based, 1-g/kg IV, repeated every 4 to 6 h. Major complications associated with mannitol therapy include hypovolemia, hypotension, and nephrotoxicity due to impaired mannitol clearance. The osmole gap is a sensitive method for monitoring mannitol clearance using the formula: calculated osmolarity = 1.86(Na + K) + glucose/18 + BUN/2.8 [196]. Moreover, hypertonic saline has a higher reflection coefficient than mannitol, which is effective at reducing cerebral edema from both the injured and uninjured brain. Up to 30 mL boluses of 23.4% saline may be used, and a sodium level up to 160-mmol/L may be acceptable. However, sodium levels > 160-mmol/L may lead to detrimental outcomes with the development of delirium and seizures. Prophylactic osmotherapy should be avoided as it may increase the infarct volume.

Pentobarbital has shown to be effective in reducing intracranial pressure by lowering the cerebral metabolic rate and, as a free radical scavenger, through its neuroprotective property [197]. Major complications associated with barbiturate therapy are hypotension, sedation, and increased risk of infection. As there are no randomized controlled trials of barbiturate therapy in cerebral infarction, current use of pentobarbital is not recommended.

Hyperventilation decreases cerebral blood volume almost immediately and subsequently reduces intracranial pressure within minutes. As carbon dioxide is a potent cerebral vasodilator, decreasing pCO2 leads to vasoconstriction. The effect is short-lived, with the risk of rebound vasodilatation and worsening intracranial pressure when the pCO2 returns to normal and, thus, worsens the cerebral infarction volume [198].

Temperature modulation is an important component in the management of malignant cerebral infarction. Increased body temperature is strongly associated with an increased intensive care unit and overall hospital length of stay, higher mortality, and worse overall outcome [199]. Increased excitotoxicity, cell depolarization, enzymatic dysfunction, and blood–brain barrier breakdown can worsen infarct volumes [200,201]. Induced hypothermia protocols post-stroke continue to be investigational, but have shown promise in the clinical setting, warranting further study [202].

Decompressive craniectomy is effective in patients with very large infarct volumes, such as an MRI DWI volume of ≥145-cm^3^ [203]. Decompressive craniectomy has shown to reduce mortality without increasing disability. In a pooled analysis of three randomized controlled trials (DESTINY, DECIMAL, and HAMLET) [204] with similar inclusion criteria and primary outcome measures, decompressive craniectomy performed within 48 h of stroke onset was shown to reduce mortality and increase the number of patients with favorable functional outcomes. Moreover, as per the 2008 European Stroke Organization guidelines, decompressive craniectomy in patients ≤60-years of age within 48 h post-stroke significantly reduces mortality compared with the best medical treatment and should be sought in patients with an evolving malignant cerebral infarction [174].

In the past, guidelines for decompressive craniectomy were not well-established. Some groups would await herniation prior to decompressive craniectomy, while others would proceed to decompressive craniectomy within 24 h, based on the infarct size alone [205]. However, the 2019 AHA/ASA guidelines for the early management of AIS provide general recommendations for the medical and surgical management of malignant cerebral infarction [41].

### 9.3. Posterior Circulation Large Vessel Occlusion

Posterior circulation AIS occurs as a clinical syndrome secondary to stenosis, thrombosis, embolism and/or thromboembolism within the posterior circulation, comprised of the extracranial and intracranial vertebral, basilar, and posterior cerebral arteries. The most common etiology in posterior circulation strokes is occlusion or thromboembolism from vertebrobasilar atherosclerosis and/or dissection and cardioembolism [206,207].

In acute basilar occlusion, EVT should be considered as there is a high likelihood of death or severe disability when recanalization is not achieved [208,209,210]. The treatment window for IV TPA and/or EVT may be extended beyond 4.5 h, and up to 24 h from symptom onset, based on clinical judgement [211]. The extended treatment window is likely related to having a higher proportion of white matter in the brainstem and more robust collaterals making it more resistant to ischemia [211]. Thus, there is more salvageable tissue within the posterior circulation, which may exist well beyond the time window for anterior circulation stroke thrombolysis. Thrombolysis in posterior circulation strokes has been shown to be particularly effective in patients with stuttering symptoms, with no radiological evidence of extensive infarction [212]. Moreover, in the BASILAR study [213], patients with acute basilar artery occlusion were found to have better functional outcomes and reduced mortality when EVT was performed within 24 h of their last known normal, while in another trial of EVT for basilar artery occlusion [214], there was no significant difference found in favorable functional outcomes between intervention and medical therapy. However, in an accompanying editorial [215], potential bias in the selection process may have influenced the results. Similarly, the BEST trial on EVT versus standard medical therapy for vertebrobasilar artery occlusion found no significant difference in the favorable outcomes of patients receiving EVT compared to those receiving standard medical therapy alone [216].

Emergency angioplasty, or the stenting of the extracranial vertebral arteries, has not been well-established in this clinical setting [217]. A study reported that stenting for symptomatic vertebral artery stenosis had a much higher risk for intracranial than extracranial stenosis, while this pooled analysis did not show any benefit for stroke prevention with either intracranial or extracranial treatment [218].

External ventricular drainage or posterior fossa decompression, with partial removal of the infarcted tissue, are neurosurgical interventions that may be lifesaving in large volume cerebellar infarction with a declining level of consciousness attributable to raised intracranial pressure or acute hydrocephalus [41,219,220].

## 10. Conclusions

Stroke management has been a rapidly evolving discipline of neurology. There have been better delineations of distinct stroke entities, which have helped stratify and optimize management approaches. Therapeutic stroke management has greatly expanded beyond the utility of IV TPA, with more advanced and complex techniques. EVT has revolutionized stroke management through the direct visualization of the clot and timely clot retrieval techniques improving patient outcomes. There is convincing evidence that stroke prevention techniques continue to decrease the risk of recurrent strokes. Stroke preventive measures have expanded, with substantial evidence for alternate antiplatelet therapies and oral anticoagulants, which have helped improve patient management. The major underpinning of high-quality stroke care is multidisciplinary care, which initiates from the prehospital setting, and continues well beyond stroke rehabilitation.

In this article, we have discussed the evaluation and the current advances in the management of AIS, starting in the prehospital setting and in the emergency department, followed by post-acute stroke hospital management and rehabilitation. We have highlighted special scenarios in stroke care where practice gaps currently exist, warranting further studies. Despite paradigm shifts in stroke management, post-stroke disability remains pervasive. Further studies are warranted in developing effective prehospital systems for the rapid identification and transfer of patients to appropriate stroke centers for timely management with thrombolytics, as well as establishing the role for EVT in posterior circulation and distal vessel occlusions.

## Figures and Tables

**Table 1:** National Institutes of Health Stroke Scale.

	Category	Description	Score
1A	Level of consciousness	Alert	0
		Drowsy	1
		Stuporous	2
		Coma	3
1B	Level of consciousness—questions	Answers both correctly	0
		Answers one correctly	1
		Answers both incorrectly	2
1C	Loss of consciousness—commands	Performs both tasks correctly	0
		Performs one task correctly	1
		Performs neither task correctly	2
2	Best gaze	Normal	0
		Partial gaze palsy: can be overcome	1
		Partial gaze palsy: corrects with oculocephalic reflex	1
		Forced gaze palsy: cannot be overcome	2
3	Visual fields	No visual loss	0
		Partial hemianopia	1
		Complete hemianopia	2
		Bilateral hemianopia	3
4	Facial Palsy	Normal symmetric movements	0
		Minor paralysis	1
		Partial paralysis	2
		Complete paralysis of one or both sides	3
5A	Left Arm Motor Drift	No drift	0
		Drift	1
		Some effort against gravity	2
		No effort against gravity	3
		No movement	4
5B	Right Arm Motor Drift	No drift	0
		Drift	1
		Some effort against gravity	2
		No effort against gravity	3
		No movement	4
6A	Left Leg Motor Drift	No drift	0
		Drift	1
		Some effort against gravity	2
		No effort against gravity	3
		No movement	4
6B	Right Leg Motor Drift	No drift	0
		Drift	1
		Some effort against gravity	2
		No effort against gravity	3
		No movement	4
7	Limb Ataxia	Absent	0
		Present in one limb	1
		Present in two limbs	2
8	Sensation	Normal; no sensory loss	0
		Mild-to-moderate sensory loss	1
		Severe-to-total sensory loss	2
9	Best Language	No aphasia; normal	0
		Mild-to-moderate aphasia	1
		Severe aphasia	2
		Mute; global aphasia	3
10	Dysarthria	Normal	0
		Mild-to-moderate dysarthria	1
		Severe dysarthria	2
11	Extinction/Inattention	No abnormality	0
		Visual, tactile, auditory, spatial, or personal inattention	1
		Profound hemi-inattention or extinction	2
Total Score = 0 to 42			

**Table 2:** Stroke Mimics.

Hypoglycemia
Seizures
Syncope
Brain tumor
Primary headache disorder (e.g., hemiplegic migraine)
Toxic, metabolic, hypertensive encephalopathy
Sepsis
Recreational drugs and alcohol use
Benign paroxysmal positional vertigo
Subdural hematoma
Transient global amnesia
Dementia
Demyelinating disease
Neuropathy
Myasthenia gravis
Bell’s palsy
Conversion disorder

**Table 3:** Indications and Contraindications of Intravenous Recombinant Tissue Plasminogen Activator.

Indications	Additional Recommendations	Contraindications
Mild disabling to severe stroke symptoms Symptom onset ≤ 4.5 h prior to treatment Age ≥ 18 years Blood pressure < 185/110 mm Hg Blood glucose > 50 mg/dL NCCT demonstrates early ischemic changes of mild to moderate extent other than frank hypodensity Prior antiplatelet therapy, either monotherapy or dual antiplatelet therapy End-stage renal disease; normal aPTT	Age > 80 years presenting in the 3- to 4.5-h window History of prior stroke and diabetes mellitus presenting in the 3- to 4.5-h window Mild disabling and severe (NIHSS > 25) stroke symptoms presenting in the 3- to 4.5-h window Wake-up and unknown time of onset with MRI-DWI lesion smaller than one-third of the MCA territory with no visible signal change on FLAIR Preexisting disability (mRS score ≥ 2) or dementia Early improvement of symptoms with persistent moderate impairment Seizure at onset and if residual impairments are secondary to stroke and not postictal changes Initial blood glucose < 50 or > 400 mg/dL that are normalized with persistent symptoms Coagulopathy—history of warfarin use with INR ≤ 1.7 or PT < 15 s Dural puncture Arterial puncture of a noncompressible blood vessel Recent major trauma within 14 days not involving the head Recent major surgery within 14 days Prior gastrointestinal and genitourinary bleeding Menstruation without a history of menorrhagia Extracranial cervical dissection Intracranial arterial dissection Small to moderate-sized unruptured and unsecured intracranial aneurysm (<10 mm) Intracranial vascular malformation Few cerebral microbleeds (≤10) Concomitant tirofiban, eptifibatide Extra-axial intracranial neoplasm Acute myocardial infarction Recent myocardial infarction within 3 months *** Acute pericarditis Left atrial or ventricular thrombus Other cardiac disease (e.g., cardiac myxoma, papillary fibroelastoma) Procedural stroke (e.g., cardiac or cerebral angiography) Systemic malignancy with >6-month life expectancy without other contraindications Pregnancy Ophthalmologic conditions Sickle cell disease Hyperdense MCA sign Illicit drug use Stroke mimics	Mild nondisabling stroke symptoms (NIHSS score 0–5) presenting within 4.5 h of symptom onset NCCT demonstrates extensive regions of clear hypoattenuation Intracranial hemorrhage Prior ischemic stroke within 3 months Severe head trauma within 3 months Acute head trauma Intracranial or intraspinal surgery within 3 months History of intracranial hemorrhage Symptoms and signs consistent with subarachnoid hemorrhage Structural gastrointestinal malignancy or recent bleeding within 21 days Coagulopathy * ○ Platelets < 100,000/mm^3^ ○ INR > 1.7 ○ aPTT > 40 s ○ PT > 15 s Treatment with full dose low molecular weight heparin within 24 h Treatment with thrombin inhibitors or factor Xa inhibitors within 48 h ** Concomitant abciximab Concomitant intravenous aspirin Infective endocarditis Aortic arch dissection Intra-axial intracranial neoplasm

Abbreviations: aPTT = activated partial thromboplastin time; DWI = diffusion weighted imaging; FLAIR = fluid attenuation imaging recovery; INR = international normalized ratio; IV TPA = intravenous recombinant tissue plasminogen activator; MCA = middle cerebral artery; MRI = magnetic resonance imaging; NCCT = non-contrast computed tomography; mRS = modified Rankin Scale; NIHSS = National Institutes of Health Stroke Scale; PT = prothrombin time; STEMI = ST-elevation myocardial infarction. * In patients without a history of thrombocytopenia, treatment with IV TPA can be initiated while awaiting the results of platelet count but should be discontinued if platelet count is <100,000/mm^3^. Similarly, in patients without recent use of oral anticoagulants or heparin, IV TPA can be initiated while awaiting the results of coagulation tests but should be discontinued if INR is >1.7 or PT is abnormally elevated as per the laboratory standards. ** IV TPA could be considered when coagulation tests (e.g., aPTT, INR, platelet count, ecarin clotting time, thrombin time, direct factor Xa activity assays) are normal or when the patient has not received a dose of an anticoagulant for >48 h with a normal renal function. *** Recent myocardial infarction within 3 months: reasonable to treat the ischemic stroke with IV TPA if the recent myocardial infarction was a non-STEMI, STEMI involving the right or inferior myocardium, or STEMI involving the left anterior myocardium.

**Table 4:** Landmark Trials of Endovascular Thrombectomy in Acute Ischemic Stroke.

Trials	N=	NIHSS	Premorbid Condition	Treatment Window (h)	Treatment vs. Control Arms	Territory of Vessel Occlusion	Neuroimaging & Selection Criteria	TICI 2b/3 (%)	Symptomatic ICH (%)	90-Days mRS 0–2 (%)	Mortality (%)
MR CLEAN [97]	502	≥2	None	≤6	IA UK/TPA/device + IV TPA vs. IV TPA	ICA, M1, M2, A1, or A2	CT, MRI, CTA, MRA, DSA No criteria	58.7	7.7 vs. 6.4	32.6 vs. 19.1 (*p* < 0.05)	18.9 vs. 18.4
ESCAPE [98]	316	>5	Barthel index ≥ 90	≤12	Stent retriever + IV TPA vs. IV TPA	MCA trunk and immediate branches and intracranial ICA	NCCT, mCTA Small infarct core (ASPECTS 6–10); Good-to-moderate collateral flow	72.4	3.6 vs. 2.7	53 vs. 29.3 (*p* < 0.001)	10.4 vs. 19
SWIFT PRIME [94]	196	8–29	mRS 0–1	≤6	Stent retriever + IV TPA vs. IV TPA	Intracranial ICA, M1, or both	NCCT, CTA, MRA, MRI-DWI, CTP, MRP Small infarct core (ASPECTS > 5)	88	0 vs. 3	60 vs. 35 (*p* < 0.001)	9 vs. 12
EXTEND-IA [96]	70	None	mRS 0–1	≤6	Stent retriever + IV TPA vs. IV TPA	ICA or MCA	NCCT, CTA, CTP Evidence of salvageable tissue and ischemic core < 70 mL on CTP	86	11 vs. 15	71 vs. 40 (*p* = 0.01)	9 vs. 20
REVASCAT [95]	206	≥6	mRS 0–1	≤8	Stent retriever + IV TPA vs. IV TPA	Intracranial ICA or M1	NCCT, MRI-DWI Absence of large ischemic core; ASPECTS >7 on NCCT or >6 on MRI DWI	65.7	1.9 vs. 1.9	44 vs. 28 (OR 2.1)	19 vs. 16
DAWN [100]	206	≥10	mRS 0–1	≤24	Trevo retriever + IV TPA vs. IV TPA	Intracranial ICA and/or MCA-M1	NCCT, MRI, MRA, CTA, MRI-DWI, CTP <1/3 MCA territory involved Clinical Imaging Mismatch: Group A—Age ≥ 80, NIHSS ≥ 10, infarct volume < 21 mL Group B—Age < 80, NIHSS ≥ 10, infarct volume < 31 mL Group C—Age < 80, NIHSS ≥ 20, infarct volume 31–51 mL.	84—Modified TICI ≥ 2b 72.6—Original TICI ≥ 2b 10.4—TICI 3	6 vs. 3	49 vs. 13 (PPS > 0.999); Utility-weighted mRS mean score—5.5 vs. 3.4 (PPS > 0.999); NNT—2 and 2.8 for lower disability and functional independence, respectively	19 vs. 18
DEFUSE-3 [101]	182	≥6	mRS 0–2	≤16	Trevo Retriever/Solitaire device/Penumbra system + IV TPA vs. IV TPA	ICA or M1; Cervical or intracranial carotid occlusions with or without tandem MCA lesion	CTA, MRA, CTP, MRP, MRI-DWI Target Mismatch Profile: Infarct volume (ischemic core) < 70 mL Ratio of volume of ischemic tissue to infarct volume of ≥1.8 Absolute volume of potential reversible ischemia (penumbra) ≥ 15 mL	13—TICI 2a 57—TICI 2b 19—TICI 3	7 vs. 4	45 vs. 17 (OR 2.67; *p* < 0.001)	14 vs. 26

Abbreviations: A1 = first segment of anterior cerebral artery; A2 = second segment of anterior cerebral artery; ASPECTS = Alberta Stroke Program Early Computed Tomography Score; CT = computed tomography; CTA = computed tomography angiography; CTP = computed tomography perfusion; DSA = digital subtraction angiography; DWI = diffusion weighted imaging; IA = intra-arterial; ICA = internal carotid artery; IV TPA = intravenous recombinant tissue plasminogen activator; M1 = first segment of middle cerebral artery; M2 = second segment of middle cerebral artery; MCA = middle cerebral artery; mCTA = multiphasic computed tomography angiography; MRA = magnetic resonance angiography; MRP = magnetic resonance perfusion; mRS = modified Rankin Scale; NCCT = non-contrast computed tomography; NIHSS = National Institutes of Health Stroke Scale; NNT = number-needed-to-treat; OR = odds ratio; PPS = posterior probability of superiority; TICI = thrombolysis in cerebral infarction; UK = urokinase.

## Data Availability

Not applicable.
